# Longitudinal data on employee attitudes and personality in activity-based work environments

**DOI:** 10.1016/j.dib.2025.111382

**Published:** 2025-02-14

**Authors:** Freyr Halldorsson, Halldor Valgeirsson, Kari Kristinsson

**Affiliations:** a Department of Business and Economics, Reykjavik University, Menntavegur 1, 102 Reykjavik, Iceland; b School of Business, University of Iceland, Sæmundagata 2, 102 Reykjavik, Iceland

**Keywords:** Office design, Employee perceptions, Employee outcomes, Individual differences

## Abstract

Longitudinal data were collected from two smaller organizations implementing activity-based work environments. The sample consisted of a total of 50 employees, with each employee being surveyed four times. The initial survey took place a month before the activity-based work environment was implemented, followed by measures one, six, and 12 months after implementation. At each measurement point, the response rate was 58–76 %, providing a total of 130 observations. All data were collected using an online survey, with participants receiving a link via email followed up with two reminders. Each survey contained the same questions, except for the personality items, which were only included in the first survey. Participation was voluntary, and participants consented to take part. No incentives were provided for participating.

Specifications Table

**Table:** 

Subject	Applied Psychology
Specific subject area	Employee attitudes and personality and the effects of implementing an activity-based work environment.
Type of data	Table (Excel)
Data collection	Data were collected by surveying employees at four time-points, using online surveys administered via Alchemer.com (previously SurveyGizmo.com). The initial survey took place a month before activity-based work environments were implemented, followed by measures one, six, and 12 months after implementation. Each time, employees received a link via email, inviting them to participate, followed by two reminders.
Data source location	Institution: Reykjavik University City/Town/Region: Reykjavik Country: Iceland Latitude and longitude: 64.123753, −21.926866
Data accessibility	Halldórsson, Freyr; Valgeirsson, Halldor; Kristinsson, Kari (2024), “Longitudinal Data on Employee Attitudes and Personality in Activity-Based Work Environments”, Mendeley Data, V4, doi: 10.17632/ykdvcw248r.4 Repository name: Mendeley Data Data identification number: 10.17632/ykdvcw248r.4 Direct URL to data: https://data.mendeley.com/datasets/ykdvcw248r/
Related research article	F. Halldorsson, K. Kristinsson, H. Valgeirsson, The Importance of Employee Attitude in Activity-Based Work Environments and the Potential Role of Personality, Ergonomics, (2024), 1–13. https://doi.org/10.1080/00140139.2024.2337065

## 1 Value of the Data

• The dataset provides an opportunity to further explore the impact of activity-based work environments. Examining how such environments may affect various employee outcomes, including job satisfaction, productivity, collaboration, and communication, is possible.
• The study's longitudinal design can offer insights into the dynamics of change in the work environment over time. This allows for more decisive conclusions about the causal impacts of workspace changes on employee outcomes.
• The data may be of interest beyond organizational psychology in fields like ergonomics, architecture, and business management. The data may support an interdisciplinary view of the interface between workplace design, employee behavior, and organizational strategy.
• The dataset can aid in the testing and refinement of theoretical frameworks related to workplace design and its impact on employees. For example, it could be applied to help advance theories about psychological ownership, privacy perceptions, and their correlations with employee outcomes in activity-based work environments.
• The dataset can help inform best practices for designing activity-based workspaces that enhance employee engagement and efficiency, providing insights for organizational leaders and designers.

## 2 Background

The primary motivation for collecting the data stems from the increasing prevalence of activity-based work environments [1] and an interest in further supporting and expanding upon previous findings that highlight the importance of employee attitudes in successfully implementing these environments [2], as such attitudes directly influence privacy perceptions and psychological ownership [2].

The data was collected in Iceland from two organizations: (A) the administrative office of a trade union and (B) a municipally owned organization providing social housing. The data was collected to further establish employee attitude toward an activity-based work environment as an essential variable and examine if individual differences (i.e., personality traits) may predict such attitudes. Given the small sample size, the data provides valuable insights but should be regarded as an exploratory contribution rather than a foundation for broad generalizations. However, the findings offer meaningful preliminary insights into the relationship between individual differences and attitudes toward activity-based work environments, paving the way for more comprehensive studies.

## 3 Data Description

The data consists of responses from employees of two smaller organizations in Iceland moving to activity-based work environments. All employees in both organizations were included in the sample. Potential participants were 50 (Organization A had 28 employees; Organization B had 22). The data was collected with an online survey, and three reminders were sent to employees to encourage survey completion, ensuring a higher response rate and better representation of the organizations studied.

At each measurement point, the response rate was 58–76 %, providing 130 observations over 13 months. Specifically, 38 participants responded at time 1; of those, 29 responded at time 2, 31 at time 3, and 32 at time 4. Since the same participants were surveyed repeatedly, the data structure is hierarchical, with multiple measurements nested within each participant.

Before moving to a new location with an activity-based layout, employees of both organizations had assigned workstations primarily in private offices or shared with one or two others. However, a few employees had workstations in an open-plan layout. After the move, employees did not have assigned workspaces. Instead, they could choose from several types of work areas, including workstations in an open plan, small break-out/multi-purpose rooms, workstations in quiet zones, lounge areas, and formal meeting rooms.

The data is publicly available in the Mendeley Data repository through the link provided above, under Data Accessibility. An Excel file (“Survey-Items.xlxs”) includes a list of all survey items. Three questions are reverse worded and are indicated with an ‘R’ in column D. Descriptive statistics for the scales across each measurement point can be seen in Table 1, including the number of observations (n), minimum and maximum values, means, and standard deviations.

**Table 1:** Descriptive statistics for scales across measurement points.

*Variable*	*n*	*Min.*	*Max.*	*Mean*	St*.dev.*
Job performance (self-rated)—T1	38	2.5	5.00	4.15	.54
Job performance (self-rated)—T2	29	2.8	5.00	4.06	.55
Job performance (self-rated)—T3	32	3.00	5.00	4.21	.47
Job performance (self-rated)—T4	32	3.5	5.00	4.34	.40

Privacy—T1	36	1.33	5.00	3.48	.91
Privacy—T2	28	1.33	4.83	3.23	.93
Privacy—T3	30	1.00	5.00	3.28	1.09
Privacy—T4	32	1.50	4.83	3.38	1.06

Psychological ownership—T1	36	1.00	5.00	3.85	1.06
Psychological ownership —T2	28	1.00	5.00	2.90	1.20
Psychological ownership —T3	28	1.00	5.00	3.13	1.25
Psychological ownership —T4	30	1.00	5.00	3.31	1.44

Job satisfaction—T1	38	1.67	5.00	3.98	.83
Job satisfaction —T2	27	1.33	5.00	3.90	.84
Job satisfaction —T3	31	1.33	5.00	3.94	.89
Job satisfaction —T4	32	2.67	5.00	4.16	.72

ABW-attitude—T1	38	1.00	5.00	3.87	.99
ABW-attitude —T2	29	2.00	5.00	4.17	.85
ABW-attitude —T3	32	3.00	5.00	4.00	.72
ABW-attitude —T4	32	2.00	5.00	4.06	.84

Collaboration—T1	38	1.67	5.00	3.90	.66
Collaboration—T2	29	2.33	5.00	4.03	.66
Collaboration—T3	32	2.00	5.00	3.98	.69
Collaboration—T4	32	1.00	5.00	4.07	.86

Communication—T1	38	1.67	5.00	3.80	.77
Communication —T2	29	2.33	5.00	3.89	.61
Communication —T3	32	2.33	5.00	3.97	.75
Communication —T4	32	2.00	5.00	3.96	.80

Organizational commitment—T1	38	1.67	5.00	4.12	.82
Organizational commitment—T2	27	2.33	5.00	4.04	.76
Organizational commitment—T3	31	1.33	5.00	4.12	.86
Organizational commitment—T4	32	2.33	5.00	4.23	.79

P-E fit/Task-E fit—T1	35	1.00	5.00	3.75	1.11
P-E fit/Task-E fit—T2	27	1.00	5.00	3.40	1.24
P-E fit/Task-E fit—T3	31	1.00	5.00	3.55	1.37
P-E fit/Task-E fit—T4	31	1.00	5.00	3.54	1.38

Turnover intentions—T1	38	1.00	5.00	2.10	1.09
Turnover intentions—T2	29	1.00	5.00	2.22	1.07
Turnover intentions—T3	32	1.00	5.00	2.04	1.09
Turnover intentions—T4	32	1.00	5.00	1.97	.99

Work environm. satisfaction—T1	38	2.25	4.63	3.44	.58
Work environm. satisfaction —T2	29	2.50	5.00	3.86	.77
Work environm. satisfaction —T3	32	2.50	5.00	4.01	.74
Work environm. satisfaction —T4	32	1.75	5.00	3.96	.83

An Excel file (“Data.xls”) contains the raw data for all variables across the three measurement points.

## 4 Experimental Design, Materials and Methods

The data was obtained from two organizations, with data collection starting in early 2020 and lasting about 13 months.

Data were collected four times, with the first survey administered a month before moving to the activity-based work environment. The second survey took place a month after moving, with additional measures six and 12 months later. The survey was administered using an online survey tool (Alchemer.com). At each time, potential participants received an email with a brief description and a link to the study, followed up with two reminders.

Before responding to any items, all participants provided consent for their participation. It was noted that participation was voluntary and that participants could withdraw without consequences. It was also stated that participants could skip any items if they did not want to respond. Participants were promised anonymity with a random numeric code used to link data over time. No incentives for participating were provided.

The survey included various measures, including psychological ownership, privacy, work environment satisfaction, job satisfaction, and more. Table 2 contains the measures in the survey and briefly describes each.

**Table 2:** Survey measures and descriptions.

*Measure*	*Description*
*Job performance (self-rated)*	Five items from Oldham [3]. Use a 5-point Likert scale ranging from 'strongly disagree' to 'strongly agree.'
*Privacy*	Privacy was measured using six items from Oldham [3]. The measure captures two dimensions—'task privacy' and 'communication privacy’. The construct was captured using a 5-point Likert scale ranging from 'Strongly disagree' to 'Strongly agree.'
*Psychological ownership*	Three items adapted from Brown [4]. The scale was captured using a 5-point Likert scale ranging from 'Strongly disagree' to 'Strongly agree.'
*Job satisfaction*	Two items from Veitch et al. [5], using a 5-point Likert scale ranging from 'Strongly disagree' to 'Strongly agree.'
*Attitude towards activity-based work environments (ABW-attitude)*	A single item intended to capture participants' overall attitude towards activity-based work [2]. Participants responded to the item using a 5-point Likert scale—ranging from 'Strongly disagree' to 'Strongly agree.'
*Collaboration*	Three items captured collaboration using a 5-point Likert scale ranging from 'Strongly disagree' to 'Strongly agree.'
*Communication*	Three items captured communication using a 5-point Likert scale ranging from 'Strongly disagree' to 'Strongly agree.'
*Organizational commitment*	Commitment was captured using three items adapted from Mowday et al. [6]. A 5-point Likert scale ranging from 'Strongly disagree' to 'Strongly agree' was used.
*P-E fit/Task-E fit*	Three items captured the perceived fit of the work environment using a 5-point Likert scale ranging from 'Strongly disagree' to 'Strongly agree.'
*Turnover intentions*	Three items captured collaboration using a 5-point Likert scale ranging from 'Strongly disagree' to 'Strongly agree.'
*Work environment satisfaction*	Participants were asked to rate how satisfied they were with their work environment using a 5-point Likert scale ranging from 'Very unsatisfactory' to 'Very satisfactory.' The measure includes 8 specific factors (e.g., air quality, lighting, temperature, etc.) in addition to a single overall rating of the work environment.
*Personality*	The 60 items from the HEXACO-60 inventory [7] were used to capture personality traits at time 1. The scale uses a 5-point Likert scale ranging from 'Strongly disagree' to 'Strongly agree.'
*Gender*	The gender of respondents.
*Age*	Participant age in whole years.
*Job tenure*	Respondents’ tenure in months in their current job.
*Current workspace*	Type of workspace at Time 1, i.e., before moving to the activity-based work environment.
*Organization*	A dummy variable indicating which organization participants belong to.

When the data collection was complete, all results were downloaded, and any irrelevant or potentially identifying data (e.g., timestamps or IP addresses) were removed, leaving only anonymized IDs and survey responses.

Data analysis was conducted using statistical software (Stata 18). Due to the non-independence of measurements from the same individuals across different conditions, hierarchical linear modeling (HLM) was employed to address potential analytical complications.

## Limitations

The small sample size inherently limits the complexity of the statistical models that can be applied, reducing the robustness of any conclusions drawn. Furthermore, the timing of data collection in relation to the COVID-19 pandemic likely introduced additional variability into the results. While the first survey was conducted prior to the pandemic reaching Iceland, the subsequent three measurements coincided with varying levels of government-imposed restrictions on gatherings. At times 2 and 3, limits of 100 and 200 people were in place, which may have affected workplace dynamics but still allowed for some level of normal interaction. By time 4, however, the restriction had tightened to a mere 20 individuals, meaning that many employees could not physically be present at work simultaneously. This shift in workplace conditions likely influenced employees' perceptions and experiences, potentially amplifying concerns about collaboration, communication, and the functionality of activity-based work environments. Such unique contextual factors may have shaped employee responses in ways that differ from non-pandemic scenarios, adding a layer of complexity to the interpretation of the findings. Additionally, all data collected is based on self-reported responses from employees, which might introduce social desirability bias. Although anonymity was assured in our study, future research could benefit from incorporating more indirect measures or including social desirability scales.

## Ethics Statement

The study met all local ethics standards, and all participants provided their consent before taking part. According to Iocal laws and regulations, researchers must only apply for ethics approval when conducting research involving deceit, collecting health-related data, or working with vulnerable individuals or groups (e.g., children and individuals who have difficulty assessing risk and providing informed consent). This does not apply to the current data, so no ethics approval number is available.

## CRediT author statement

**Freyr Halldorsson:** Supervision, Conceptualization, Methodology, Data Curation, Writing- Original draft preparation, Writing- Reviewing and Editing. **Kari Kristinsson:** Supervision, Methodology, Writing- Reviewing and Editing. **Halldor Valgeirsson:** Supervision, Visualization, Investigation, Software.

## Data Availability

Mendeley DataLongitudinal Data on Employees in ABW Environments (Original data).
